# How do third sector organisations use research and other knowledge? A systematic scoping review

**DOI:** 10.1186/s13012-015-0265-6

**Published:** 2015-06-06

**Authors:** Rebecca Hardwick, Rob Anderson, Chris Cooper

**Affiliations:** CLAHRC for the South West Peninsula, University of Exeter Medical School, University of Exeter, Exeter, UK; Institute of Health Research, University of Exeter Medical School, University of Exeter, Exeter, UK

**Keywords:** Knowledge translation, Translational research, Implementation science, Research use, Health services, Social care, Third sector, Review

## Abstract

**Background:**

Third sector organisations (TSOs) are a well-established component of health care provision in the UK’s NHS and other health systems, but little is known about how they use research and other forms of knowledge in their work. There is an emerging body of evidence exploring these issues but there is no review of this literature. This scoping review summarises what is known about how health and social care TSOs use research and other forms of knowledge in their work.

**Methods:**

A systematic search of electronic databases was carried out with initial exploratory searching of knowledge mobilisation websites, contacting authors, and hand searching of journals. The literature was narratively summarised to describe how TSOs use knowledge in decision making.

**Results:**

Ten qualitative and mixed methods studies were retrieved. They show that TSOs wish to be “evidence-informed” in their decision making, and organisational context influences the kinds of research and knowledge they prefer, as well as how they use it. Barriers to research use include time, staff skill, resources and the acontextual nature of some academic research. Appropriate approaches to knowledge mobilisation may include using research intermediaries, involving TSOs in research, and better description of interventions and contexts in academic publications to aid applying it in the multi-disciplinary contexts of TSOs. TSOs identified specific benefits of using research, such as confidence that services were good quality, ability to negotiate with stakeholders and funders, and saving time and resources through implementing interventions shown to be effective. The small number of included studies means the findings need further confirmation through primary research.

**Conclusions:**

As the contribution of health and social care TSOs to service delivery is growing, the need to understand how they mobilise research and other forms of knowledge will continue. The research community could 1) develop relationships with TSOs to support the design and development of research projects, 2) use a range of methods to evaluate interventions to facilitate TSOs applying them to their organisational contexts and 3) improve our understanding of how TSOs use knowledge, through the use of complementary research methods, such as a realist review or ethnography.

**Electronic supplementary material:**

The online version of this article (doi:10.1186/s13012-015-0265-6) contains supplementary material, which is available to authorized users.

## Background

Third sector organisations (TSOs) play an important and expanding role in health and social care provision in the UK, Canada and many other developed countries [1–3]. TSOs can be broadly defined as organisations which are formally organised; non-profit distributing; constitutionally independent from the state; self-governing and benefiting from some form of voluntarism (e.g. with volunteer (unpaid) Trustees or Board members or using volunteers in the delivery of services) [4]. This definition of TSOs includes what were previously, or are elsewhere, called voluntary, charitable or community-based organisations (CBOs) [2, 3, 5]. In 2011, there were estimated to be over 35,000 TSOs providing health and social care services in England [6]. In the UK, income for TSOs from public-sector contracts and grants has increased from £9.1 billion in 2001/2 to £14.2 billion in 2010/11 [7], and since 2006, the proportion of NHS expenditure on health services purchased from TSOs has increased year-on-year (source: response by Department of Health to Freedom of Information request by authors, December 2013).

TSOs are also believed to have particular strengths, relative to public sector organisations. These claimed strengths include the following: being more client-led and community-led; able to access “hard to reach groups”; being responsive to local people; being innovative, builders of social capital and civil participation; more cost-effective and; more approachable and less threatening as service providers [2, 3, 8, 9]. Many of these strengths and differences are embedded in the way they work (person-centred, participatory flexible). As knowledge mobilisation is increasingly regarded as an inherently social process [10, 11], affected by contextual enablers and constraints, it is likely that these particular strengths and differences will affect how and why they mobilise knowledge in their work. For instance, TSOs tend to work with whole communities, whose needs are diverse and so research evidence which is narrowly focused on a particular intervention at a particular point in time, may have less meaning and utility than other ways of knowing (such as peer-to-peer learning, “borrowing ideas” from other organisations or using staff and service users’ tacit knowledge).

Despite indicative evidence that the distinctive cultures and objectives of TSOs may mean that they mobilise knowledge differently [12], compared with research into knowledge mobilisation and research use in public sector service organisations, there seems to be relatively little equivalent research in TSOs working in health and social care. It is important to know how TSOs mobilise knowledge in their work, if the kinds of functions they carry out (service delivery, advocacy and capacity building) are to be good quality, safe and effective but, as of yet, a review of available evidence on this topic has not been undertaken.

This paper presents the findings of a scoping review to answer the following questions:

1. what research evidence is currently available about how TSOs that provide health and social care services use research and other forms of knowledge in decision making?
2. what are the implications of this research for the research community, as well as TSOs themselves?

## Methods

Scoping reviews have been defined as “a form of knowledge synthesis that addresses an exploratory research question aimed at mapping key concepts, types of evidence, and gaps in research related to a defined area or field by systematically searching, selecting and synthesizing existing knowledge’” [13]. It includes the following steps: identifying the research question; identifying relevant studies; study selection; charting the data (data extraction); collating, summarising and reporting the results and finally consultation on findings with stakeholders. In this scoping review, steps 1–5 were carried out by the authors; step 6 was omitted due to the confines of time and resources. Assessing the quality of included studies is not typically carried out in a scoping review [13, 14], and so no formal quality assessment was undertaken.

### Searches

The literature searching for this review occurred in the following two phases: (1) an exploratory search, including bibliographic database browsing, web-searching, contacting authors and experts and hand-searching of relevant journals, the results of which informed (2) a formal systematic search of a wider range of bibliographic databases and other e-resources.

The exploratory search combined third sector organisational terms (e.g. charity, voluntary, community-based) and terms related to knowledge mobilisation (e.g. knowledge transfer, knowledge exchange, research utilisation) within the PubMed database. It was initially carried out to inform a funding application. Further searching was undertaken of websites of knowledge mobilisation organisations in the UK and Canada, contacting authors and hand-searching of relevant journals (*Voluntary Sector Review*, *Implementation Science*) and revealed a small body of emerging research and published commentary on how TSOs use and generate research (11 potentially includable studies) [12, 15–24].

The second bibliographic search was developed by an information specialist (CC) and employed a wider range of search terms to identify literature about TSOs and their knowledge mobilisation or research use. The search strategy was run in the following bibliographic databases of published and grey literature, from database inception to date of search: HMIC via OVID, Social Policy and Practice via OVID, CommunityWise via Oxmill, ASSIA via ProQuest, British Library Social Welfare Portal. A copy of the search strategy is in Additional file 1.

### Study inclusion and exclusion criteria

Articles were eligible for inclusion if they were in English language and were as follows:

- research studies
- of knowledge mobilisation, or research use
- conducted in third sector organisations which are involved in providing or commissioning health or social care services.

Please see the definitions and detailed inclusion/exclusion criteria in Table 1.

**Table 1:** Key definitions and study eligibility criteria

Definitions	
Knowledge mobilisation and research use	Intentional strategies for increasing or improving:
	• research or knowledge use or
	• the uptake of explicitly evidence-based practices,
	or studies of what influences decision making or practice changes (including the use of knowledge within routine organisational processes)
Third sector organisations	All organisations operating outside the formal state or public sphere that are not trading commercially for profit in the market.
	(source: Third Sector Research Centre website ‘What is the third sector?’)
	http://www.birmingham.ac.uk/generic/tsrc/about/index.aspx [accessed 22nd May 2014]
	Third sector organisations carry out a range of functions, including providing services to the public directly (either funded by public sector organisations, or through charitable giving/grant funding), lobbying and campaigning on behalf of particular interest groups, supporting and networking other third sector organisations and building capacity (such as Local Infrastructure Organisations).
Include	Exclude
English Language	
Research into knowledge mobilisation or research use in third sector organisations providing health and social care services, related to physical and/or mental health support and related functional wellbeing needs e.g. community children’s services, community services for older people and the frail elderly	Probation, criminal justice services, welfare payments and other needs-based financial support
Primary or secondary research (including systematic reviews), published in peer reviewed journals or grey literature	

In deciding whether a research study was about knowledge mobilisation or not, some studies presented a dilemma. Firstly, there were a number of studies about community-based participatory research (CBPR) in which, as the abstract of one of these studies stated, CBPR was used mainly as “a strategy to develop trust and build on the strengths of partners from various settings to address significant health issues” (p133) and where the partners commonly included both academic research teams and community organisations [25]. Such participatory or collaborative research usually involves developing relationships between one or more research institutions and one or more community-based organisations [26] and could therefore be seen as direct examples of knowledge exchange through relationship building between researchers and potential research users. Similarly, collaborative community-based “action research” can be seen as a knowledge mobilisation process that brings together services providers and service users and the public—albeit one where the processes of knowledge generation (co-production) and implementation are indistinguishable. However, as these studies focused on whole communities, where the TSOs were just one of a range of actors involved, we decided not to include them.

Secondly, we also deliberated whether studies of variation in the uptake of “evidence-based practices” should be regarded as knowledge mobilisation research. We took the view that, even if a study explicitly labelled particular practices as evidence-based, then a study which only investigated attitudes towards those treatments [27] or variations in uptake was not strictly knowledge mobilisation research. It would only be knowledge mobilisation research, we decided, if there was some investigation into the *processes* of uptake of the practice or if there was an explicit initiative to promote the implementation of the evidence-based practice (e.g. Shera and Dill’s evaluation of the impact of a knowledge mobilisation strategy on engagement with evidence-informed practice [28]).

### Study selection

All articles were title and abstract screened by two of the authors (RH and RA), and those eligible for inclusion were read full text by RH and RA. Further exclusions were made at this point and any disagreements on inclusion were resolved through discussion.

### Data extraction

Two authors (RH and RA) extracted data from the final set of included studies into a data extraction table which was developed by the review authors to capture the information shown in the following:

- year of publication
- author
- title
- country where study conducted
- study aim
- methods
- type of third sector organisation
- type of services provided
- whether a specific knowledge mobilisation strategy was studied, and if so what; types of knowledge/evidence/decisions studied;
- identified barriers to knowledge mobilisation
- identified facilitators of knowledge mobilisation
- study strengths and limitations
- author-identified areas for further research.

### Data analysis

A simple thematic analysis was carried out (by RH) which mapped the range of issues the included studies raised, and to identify areas for future research. The results are presented as a narrative synthesis.

## Results

### Review statistics

**Fig. 1:**
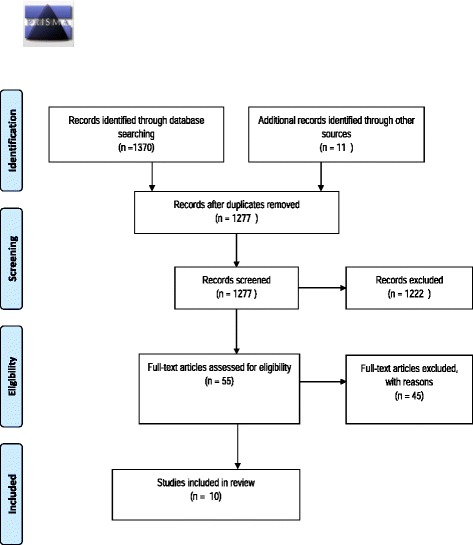
PRISMA flow diagram

The eleven records from the first search were combined with the 1370 records identified through database searching in the second search. After removing duplicates, 1277 were title and abstract screened, and 1222 were excluded as not meeting the inclusion criteria. The remaining 55 articles were retrieved as full text and read for inclusion. Of these, a further 45 were excluded as not meeting the inclusion criteria. Our review therefore included ten studies (see Fig. 1 for the study screening and selection process (PRISMA) diagram).

Six studies were from Canada, two from the USA and two from the UK. The services provided by the TSOs included in the studies concerned HIV/AIDS care [18, 19, 29–31], child welfare services [28], diabetes care [30], addictions care [15], adult mental health services [18], child mental health services [32] and social welfare and health care services [33], or was mixed across domains of health and social care.

Four studies looked at the processes of implementation of evidence-based interventions or programmes by TSOs [16, 29, 31, 32]. Four studies looked at how TSOs use research knowledge in their work and decision making [18, 28, 30, 34]. Only two of these studies focussed on specific strategies for mobilising research knowledge. These were Shera and Dill (2012) [28], who looked at the use of the “Practice and Research Together (PART)” programme to “push” research into practice by TSOs via a range of mechanisms (webinars, conferences etc.); and Beddoes et al. (2012) [34], who explored the benefits of Open Access publication to facilitating knowledge use by TSOs. Finally, two studies explored how TSOs use research alongside other forms of knowledge (tacit or experiential) in their work [15, 33]. See Table 2 for a summary of study aims, methods and the types of organisation in which the research was conducted.

**Table 2:** Characteristics of included studies

Author (Date)	Aim of research	Methods	Number/Type of organisation(s) and type of care service/client group
Paper title			
Country			
Beddoes et al., (2012)	To investigate the benefits of Open Access scholarly research outputs to TSOs	Mixed methods:	TSOs, many providing health and social care services.
*Benefits of open access to scholarly research for voluntary and charitable sector organisations*		(Rapid evidence review, scoping interviews (n = 9), online survey (*n* = 101), case studies (*n* = 10))	
England and Wales			
Dolcini et al., (2010)	To investigate how agencies are translating evidence-based interventions into practice	Qualitative:	6 agencies that were implementing one of these Evidence-Based Interventions: Healthy Relationships (living with HIV/AIDS); Safety Counts (injecting drug users); Many Men, Many Voices (for gay men of colour)
*Translating HIV interventions into practice: community-based organizations' experiences with the diffusion of effective behavioral interventions (DEBIs)*		In-depth structured interviews with executive directors, programme managers and programme implementers (*n* = 15).	
USA			
Jack, et al., (2011)	To explore:	Qualitative:	24 agencies across Canada providing addiction services to women
*Evidence-informed decision-making by professionals working in addiction agencies serving women: a descriptive qualitative study*	1) the types and sources of evidence used to inform practice-related decisions within Canadian addiction agencies serving women;	In-depth telephone interviews with decision-makers (*n* = 26)	
Canada	2) how decision makers at different levels report using research evidence;		
	3) factors that influence evidence-informed decision making.		
Kimber, et al., (2012).	To explore the process of implementation of evidence-based practice in community based organisations.	Mixed methods:	A large community-based provider of child and adolescent mental health services
*Becoming an Evidence-Based Service Provider: Staff Perceptions and Experiences of Organizational Change*		Case study, comprising of an annual questionnaire (*n* = 238 to 342 per year over four years); semi-structured interviews with staff across the organisation (*n* = 13) and observation of group meetings (not reported)	
Canada			
Lavis, J. Wilson, M. (2011)	To better understand community-based organisations, and their views of and experiences with research evidence.	Qualitative:	A representative sample of community-based organisations in Canada providing care for those with (i) HIV/AIDS, (ii) Mental health/addiction problems, and (iii) Diabetes.
*Community-based organisations and how to support their use of systematic reviews: a qualitative study*		Focus group (*n* = 31) and interviews (n = 16) with same sample of executive directors and programme managers	
Canada			
McLaughlin, et al., (2010)	To explore how decisions are made in TSOs, and how evidence informs those decisions.	Qualitative:	9 non-profit care organisations providing a wide range of social, welfare and health services
*Decision-making and evidence in direct practice*		Semi-structured interviews (*n* = 15)	
Canada			
Owczarzak, J. (2012)	To explore what factors affect how HIV prevention service providers view and implement evidence-based practice	Qualitative:	8 TSOs involved in care or preventions services related to HIV/AIDs
*Evidence-based HIV prevention in community settings: provider perspectives on evidence and effectiveness*		Semi-structured interviews with staff members (*n* = 22)	
USA			
Ramanadhan et al., (2012)	To investigate how community based organisations understand evidence-based programmes and what the barriers and facilitators are which influence their usage	Qualitative:	A number (unstated) of CBOs working with ‘underserved’ populations in Boston, Lawrence and Worcester (Massachusetts)
*Perceptions of evidence-based programs among community-based organizations tackling health disparities: a qualitative study*		Interviews with staff members (n = 6) and four focus groups (*n* = 31 participants)	
USA			
Shera, W. Dill, K. (2012)	To measure the progress and impact of PARTs activities on child welfare practice in Ontario, including a focus on TSOs engagement with evidence informed practice	Mixed methods:	37 child welfare organisations in Ontario involved in the PART (Practice And Research Together) programme
*Promoting evidence-informed practice in child welfare in Ontario: progress, challenges and future directions*		Online survey, focus groups, systematic collection and analysis of feedback from learning events	
Canada			
Wilson, et al., (2011)	To assess the capacity of CBOs in the HIV/AIDS sector to acquire, assess, adapt and apply research evidence in their work.	Quantitative & qualitative:	25 community-based organisations (with ~290 full-time equivalent employees in total) providing HIV/AIDS care services
*Community capacity to acquire, assess, adapt, and apply research evidence: a survey of Ontario's HIV/AIDS sector*		Self-assessment survey (*n* = 51)	
Canada			

### The diversity of knowledge that TSOs use to inform their work

Five studies reported that TSOs use a range of information in decision making and service delivery [15, 29, 31, 33, 34]. Sources of knowledge included staff professional experience and client views and wishes [15, 29, 31, 33]; and in some of these studies [29, 33], staff and client knowledge was preferred over other sources of knowledge. For example, Dolcini’s study [29] identified organisational culture as a barrier to the implementation of evidence-based HIV practice, insofar as it was not seen as part of the culture in CBOs to rely solely on evidence-based practice; instead, there was a preference for using their own knowledge of what works, or borrowing programmes and ideas from organisations that run similar services. Similarly, McLaughlin’s study [33] in nine non-profit care organisations in Canada reported that colleagues were felt to be the most important source of information for making decisions about client care. Interviewees described working issues out collaboratively as a team, feeling that drawing on their shared values and experiences was an efficient way to access information. Another source of information was the professional and personal, or experiential knowledge of the practitioner themselves. Such knowledge was made up of a range of reflections, previous experiences, and in some instances “gut feelings”.

Clients were also sources of information; not only what they said, but what was *unsaid*; so lack of attendance at services was seen as subtle client feedback that the service was not meeting their needs appropriately [33]. The client’s own experience was understandably important in tailoring any interventions, with the need to remain open to client needs and to adapt interventions to suit them. Internally generated knowledge (from advisory committees, service-user surveys and focus groups) was important in their work and was felt to be more influential than externally produced, “academic” knowledge. A further source of information was professional values, (e.g. their Professional Code of Ethics), and the philosophy of their organisation.

In Owczarzak’s study into evidence-based practice for HIV prevention services [31], interviewees differentiated between “book” and experiential knowledge, where book knowledge was used to support intervention implementation and experiential to challenge it. The “borrowing” of ideas from others was a source of knowledge in Jack et al.’s study [15]. They found that multiple types of evidence were used, without a clear preference for any particular sort of evidence, apart from relying more on locally collected information. Research evidence was used, along with best practice guidelines, and local programme evaluations and information from programmes underway in other areas which were seen as being “best practice”. Client need assessments, expert opinion and personal experience (of addiction and recovery), as well as individual professional experience were also used to support decision making.

### Barriers to research use and knowledge mobilisation

All but two of the studies [15, 16, 18, 29, 30, 32–34] described barriers within organisations that prevented them from fully making use of research and other knowledge. These barriers included resource constraints (lack of time, people, cost and competing priorities), organisational culture, the need but difficulties in adapting evidence-based programmes to their organisational context and problems in applying the findings of scholarly research to practice. One study described the difficulties of having staff with the time and skill to access scholarly research, assess its quality and reliability and then develop user-friendly summaries [18].

Other barriers were external to the organisation, in particular, the lack of scholarly research which was seen as relevant to the organisational or community contexts of community based, or third sector organisations [16, 30, 31, 33, 34]. In Beddoes’ study of Open Access Publication [34], they found uncertainty amongst TSOs of the value of scholarly research to their organisational contexts and that the multi-disciplinary nature of how third sector organisations work (across communities, sectors and settings) did not lend itself to the way that research is organised into specific disciplines and journals, each requiring a separate subscription by the TSO. McLaughlin’s study [33] again found that academic research was seen as irrelevant to TSOs local contexts, and it appears from Ramanadhan’s study that even when a TSO wants to adapt an evidence-based programme to make it contextually relevant, funders would often not permit these (necessary) changes [16].

Lavis and Wilson explored the utility of systematic reviews for community based organisations, and some participants reflected that there may be limitations to the knowledge from systematic reviews and problems in applying the findings to their organisational context [30]. In particular, systematic reviews which lacked detailed description of the programme or intervention were unhelpful, as was lack of detail on how and why particular programmes worked [30]. Similarly, Owczarzak’s study concluded that barriers to implementation of evidence-based interventions by community based organisations may be related to the lack of attention in such evidence-based interventions to the experiences and knowledge of CBOs themselves, their staff, and their clients (and staff knowledge of their client’s needs), and that developing implementation guidance that is more population and contextually sensitive would be valuable [31].

### Facilitators to research use and knowledge mobilisation

Several studies identified similar facilitators to research use and knowledge mobilisation [15, 16, 32–34]. These concerned developing relationships between academia and TSOs, technical guidance or assistance in implementation (in the form of manuals or experts), clear leadership, interdisciplinary working, improving access to research of different kinds, evidence that similar organisations that had successfully implemented the evidence-based programme and more relevant local research.

Ramanadhan’s study found that linking with “technical assistance” (such as programme architects, researchers and funders) to help deliver the programme, and to set outputs and outcomes, was seen as beneficial [16]. Strong relationships were developed through more participatory approaches to conducting research. In particular, they noted a need for research to include CBOs so that the community context is understood as an important factor in any intervention (rather than seen as a variable in need of “controlling”). Similarly, in McLaughlin’s study, when respondents were asked what would help uptake of research evidence, more relevant, local research was highlighted as important, as well as greater understanding of the range of clients served by the organisation.

Kimber’s study of the implementation of evidence-based practice found that respondents felt the clinical transformation was a “thoughtful and intentional” process, needing clear leadership and effective mechanisms for managing the project. Respondents reported the value of including a range of disciplines and representation from the geographical spread of the organisation as it created a varied perspective on implementation and its impacts. Similarly, Jack et al. found that interviewees reported that senior support, individual skills development, along with an identified individual with responsibility and skills to locate and appraise evidence would facilitate research use [32].

Beddoes’ study of Open Access to scholarly research for third sector organisations found that facilitators of using research included the following: more freely available ways to access research (e.g. Google scholar); the importance of intermediary bodies in synthesising evidence and providing briefings for the sector; a repository for third sector research; finding better ways to improve the interaction and information sharing between academia and TSOs to make research more relevant to their decision making. Along similar lines, McLaughlin’s study found that improving access, prompt publishing and dissemination, plain English summaries, easy to use databases and better organised and coordinated research were important to facilitate research use [33].

Jack et al. said that their interviewees felt that there needed to be evidence of successful implementation elsewhere and supported by expert opinion as well as the wider community partners. They also wanted evidence that met the stated needs of women using their services and could be implemented with minimal financial and human resource implications. They also found that if there was endorsement of formal partnerships between universities and the organisations concerned, and if findings were clearer with guidance on how to apply them to practice this would also facilitate use of research knowledge [15]. Another Canadian study found that categorising systematic reviews by the determinants of health, or topics related to treatment, care and support for specific populations would enable more relevant results to be retrieved and would increase the flexibility of searching [30].

### Strengths of TSOs in knowledge mobilisation and research use

Two studies focused on the ability of TSOs to use research and other knowledge, rather than their inability. In Owczarzak’s study [31], the author argued that previous research on implementation of evidence-based interventions by CBOs had taken a capacity building approach, focusing on what an organisation *lacked* in order to faithfully implement a Diffusion of Effective Behavioural Interventions (DEBI) programme, and to a large extent ignored the values, mission, experiences and views of the implementing organisation. Owczarzak was interested in finding out what other (i.e. positive) factors influenced implementation fidelity. The study found that CBOs recognised the value and importance of evidence-based practice for HIV prevention services, some even seeing it as central to their organisational mission and identity. However, interviewees reported a conflict between what is presented as an intervention that “works” and practitioners’ own knowledge of their clients and what “works” for them. Owczarzak found that this created ambivalence amongst staff responsible for implementation towards the programme they were meant to be implementing. Furthermore, interviewees contested funder and programme designer definitions of effectiveness and what counted as evidence of effectiveness.

Wilson et al.’s survey looked more generally at what organisations were able to do, when it came to using research in practice. They found that approximately half of the organisations surveyed felt they had capacity to apply research, and more than half felt that their organisational culture supported research use. Organisations also reported being strong at finding research through networks, websites and in grey literature.

### Motivations for knowledge mobilisation and research use

Third sector organisations reported using research in order to access a range of benefits such as improved services for clients, positive impact on staff, increased confidence in negotiating with funders and avoiding implementing programmes which do not work. For others, using research was a funding requirement. Kimber’s study of the process of implementation of a number of evidence-based practices in a large community-based provider of child and adolescent mental health services in Canada, found that changes brought about by the transformation process were seen as beneficial to clients and outweighed the disadvantages [32]. The perceived impacts of implementing evidence-based practice included increased confidence amongst practitioners in practice skills, and increased confidence in their employing organisations as a leader in healthcare service provision [32]. In Lavis and Wilson’s study, which explored the use of systematic reviews by community based organisations, they found that when participants were told what a systematic review was, they felt it would be of use to their work, in terms of being assured that all relevant research had been included, avoiding the delivery of ineffective services or interventions and enabling constructive debate with stakeholders on what interventions were useful [30]. Ramanadhan et al. found that implementing evidence-based programmes was important to organisations external to the CBO (such as funders, national agencies, researchers), and can be mandated by them in order to receive funding to provide services [16].

### Processes of knowledge mobilisation and research use

Dolcini’s study looked in depth at the process of implementing an evidence-based intervention. They used the ADAPT framework, based on Rogers’ Diffusion of Innovations theory [35], which describes a series of phases in intervention implementation (assessment, preparation and implementation) and conducted interviews with members of staff responsible for programme implementation across agencies funded by the Centers for Disease Control that were implementing an HIV/AIDS Diffusion of Effective Behavioural Interventions (DEBI) programme [29]. The study found that consultation with external stakeholders was done rarely and normally after a choice of which intervention to implement had already been made. Organisations often chose interventions without considering their specific skills and capability to deliver the intervention(s), and staff tended to be initially unfamiliar with aspects of the intervention (even after it had been selected for implementation).

Preparation for the intervention normally included recruiting new staff, however, problems with staff retention meant that organisations frequently returned to earlier stages in their implementation process to re-train and induct replacement staff. The authors suggest that one way to ensure more successful implementation and address some of the problems organisations encountered would be a two-phase funding process. In phase one, funding is released and organisations assess the needs of their client group and select an appropriate intervention, and the second phase of funding is then made available for them to adapt and implement it.

In McLaughlin’s study interviewees reported that academic knowledge mobilisation was generally the role of one individual who would conduct literature reviews to inform funding applications or new projects, rather than for day to day work and decision making. The internet was also used to find information for decision making, being seen as an efficient way to get the information quickly [33].

## Discussion

This scoping review located ten qualitative or mixed methods studies that investigated how TSOs use research and other forms of knowledge in their work. There were only two studies conducted outside Canada or the USA. The organisations studied varied in terms of their size, client groups, expertise and resources. TSOs’ existing understanding and use of research knowledge varied, and many of the studies focussed on exploring the different factors that facilitate and impede knowledge mobilisation. These included practical barriers such as costs of journal subscriptions, staff skills and time to search, access, adapt and apply research to their organisational context and a lack of time for reflective practice.

A more philosophical barrier was a rejection or ambivalence towards research that failed to take into account service user and staff expertise and knowledge. This echoes issues raised by a previous discussion of the challenges and opportunities of knowledge translation and exchange in community-based organisations [12]. As stated earlier, a particular strength of TSOs is that they are client- or community-led; however, in our review, only a few studies explored how this influenced knowledge mobilisation, or the different perceptions of what counts as “knowledge” for TSOs, even though these are likely to be critical in developing approaches to knowledge mobilisation that are effective for TSOs. What we did find was that the primacy that TSOs give to the views, needs and wishes of their clients meant that research knowledge was sometimes seen as inappropriate as it failed to take account of the circumstances of their service users. The difficulty was how to adapt either the evidence-based intervention, or how to integrate the research knowledge with practitioner and service-user experiential knowledge.

This philosophical barrier is reflected in existing debates as to what constitutes valid “knowledge” for service organisations [11] and that TSO preferences for locally or internally generated evidence over externally produced evidence are only partly due to practical limitations [12, 15, 31]. We speculate that the strengths of TSOs in partnership working is reflected by their preferences for using case studies, examples of good practice in similar organisations, and even expert opinion in decision making. In several of the studies, research outputs were seen as not as important as these other sources. We wonder if the perceived “research-practice gap” mentioned by several studies demonstrates a potentially important point for developing research-use approaches with TSOs; if research is not seen as relevant to organisational culture and client, or local contexts, then it does not carry the same importance as other sorts of (experiential) knowledge. One implication is that experiential knowledge could be more fully acknowledged in knowledge mobilisation activities, and it follows that such tacit knowledge may then require criteria to judge its trustworthiness.

In terms of the identified enablers for knowledge use by TSOs, freely available plain English research summaries or evidence syntheses could be very helpful, partly due to reducing the time needed to access and understand the evidence base. Links to external researchers and research organisations were also cited as important for similar reasons. The desire to inform and co-produce research was particularly evident and would go some way towards overcoming the philosophical barrier referred to previously.

There was less evidence on how TSO strengths in service redesign influence knowledge mobilisation. However, the two studies which examined the implementation of DEBI programmes raise an important discussion about the need for a more equitable relationship between TSOs and the “evidence base”; one centred on a mutual appreciation that without involvement in the design of effective behavioural interventions, TSOs may always “fail” to implement them faithfully. The multi-disciplinary contexts within which TSOs tend to work, the patchwork of funding they use, and the importance of service user views, means that interventions are likely to be adapted before implementation. We speculate that this indicates a need for interventions which are more open to adaptation without losing their active mechanisms. Research using theory driven approaches, such as realist evaluation or review may offer a more appropriate approach to evidence-based programme design, implementation and evaluation activity [36].

### Limitations

The relatively small number of included studies means that at this point a full systematic review is probably not warranted. The findings here are in need of further confirmation through either primary research (e.g. survey, ethnography), or an evidence synthesis which can include a wider range of research and other forms of knowledge (e.g. realist review). Most of the research was qualitative; there were few studies from the UK or Europe, and relatively few on TSOs working in key service areas such as mental health, addiction or child welfare. Furthermore, since none of the studies directly compared knowledge mobilisation in TSOs and public sector service organisations providing similar care services, we cannot make reliable claims that any of the apparent features of knowledge mobilisation in TSOs highlighted in this review are wholly unique to or more significant for TSOs.

It was not always clear whether an organisation was a third sector organisation according to our definition, due to cross-national differences in language and a lack of information about the official legal status of the organisations. During screening, we made our best attempts to ensure that the included studies met our definition of third sector organisations. For instance, we did not include social enterprises (a type of business set up to achieve a social purpose) in our definition of the third sector but may have included studies of such organisations unintentionally.

### Future research

Several of the included studies identified areas for further research. The main suggested areas were: exploration of how to develop strong relationships between TSOs and research organisations and researchers, in order to develop more relevant research; understanding in more depth why different tiers or staff groups within organisations perceive the use of research in their organisation differently; evaluating the effects of evidence use on quality of service and outcomes; incorporation of multiple types of evidence in evidence syntheses and systematic reviews to reflect the diverse contexts of the many TSOs that work across disciplines and sectors; and capacity building to enable TSOs to “acquire, assess, adapt and apply” research. As discussed previously, if tacit or experiential knowledge is to be used or trusted more frequently, it may be worthwhile for future research to explore the ways in which this kind of knowledge could be critically assessed. The organisations in the studies included in this review varied too, in terms of size, remit, staffing structure, and future research could consider what impact these differences may have on how research and other knowledge gets mobilised. Future research could also consider investigating research use amongst social enterprises that provide healthcare services, and in particular, how ex-NHS provider services that have become social enterprises approach research use. If knowledge mobilisation is an inherently cultural process, contrasting an ex-NHS culture against a general charity may provide useful insights into why being a third sector organisation influences knowledge and research use. In order to provide evidence that can be adapted more easily to different contexts, we suggest using a wider range of evaluation methods, especially those which focus on evaluating the underlying active mechanisms in an intervention or programme. We did not find studies of how TSOs and their staff “blend” empirical research with experiential knowledge and we feel this could be an important area for future research to explore as well.

More generally, we think there is a need for a baseline survey of the current experiences of research use by TSOs in the UK (similar to the Wilson et al. survey that used the ‘Is Research Working for You? Tool [18]). Different research methods may be necessary to understand the way the particular organisational cultures of TSOs impact on knowledge mobilisation [37]. More recently, investigations of knowledge mobilisation are using or proposing ethnographic methods and realist evaluation to do this [37–42]; the rationale being that understanding how a “hidden” process of knowledge mobilisation occurs, and why, and who it occurs for is not something which can be easily captured in a questionnaire; knowledge mobilisation is a social process, embedded in the cultures, language and norms of organisations, groups and individuals. Therefore, research that focusses on explaining who, how, why and in what circumstances different sorts of knowledge (research, “tacit” or other) are preferred and get used, may be useful in designing approaches to knowledge mobilisation that are acceptable and more effective.

## Conclusions

This review identified a small body of literature concerning how TSOs mobilise research and other types of knowledge. The findings indicate that TSOs do use research knowledge in their work, but they appear not to privilege research above other forms of knowledge (experiential or client informed). In terms of process, there also appears to be a preference for collaborative, relational approaches to knowledge mobilisation. Third sector organisations often face financial constraints, as well as personnel time constraints which, added to the contextual nature of much research output, means that accessing, adapting and applying research knowledge in their work may be challenging. When research evidence conflicts with organisational culture, there is a preference for organisational culture, which implies that such embedded, cultural ways of working may require other sorts of “knowledge” and different strategies for implementing research-based practices into these types of organisations to inform them. Although many of the reported barriers to knowledge mobilisation may be shared with other kinds of organisations, this review suggests that because of the external, contextual and internal cultural features of most third sector organisations, the barriers may operate differently and impact differently. Research should continue to investigate the particular ways in which TSOs mobilise knowledge, in order to ensure they are able to make the best use of both research and other credible knowledge.

## Additional file

Additional file 1:
**Identification of Studies.**

## Abbreviations

CBO: community-based organisation
EBI: evidence-based intervention
EBP: evidence-based practice/programme
KM: knowledge mobilisation
TSO: third sector organisation
