# Supplementary material for: How do third sector organisations use research and other knowledge? A systematic scoping review
Source: Implement Sci. 2015 Jun 6;10:84. doi: 10.1186/s13012-015-0265-6 (PMC4530490; doi:10.1186/s13012-015-0265-6)
Supplement: Additional file 1: — Identification of Studies. [file 13012_2015_265_MOESM1_ESM.docx]

**Additional file 1: Identification of Studies**

**Bibliographic Database Searches (CC)**

**The bibliographic databases used in this scoping review were selected purposively in relation the studies aim. We also selected these databases because of the content they offer, which is a mixture of peer-reviewed studies, charity reports, grey or hard-to-locate literature, and research reports.**

| Database | # |
| --- | --- |
| HMIC | 284 |
| Social Policy and Practice | 395 |
| CommunityWise | 169 (10 imported) |
| Assia | 661 |
| British Library Social Welfare Portal | 20 |
| Total | 1370 |
| Duplicates Removed | -104 |
| Unique Records to Screen | 1266 |

Database: HMIC

Host: OVID

Data Parameters: 1979 to January 2014

Date Searched: 10/03/2014

| **#** | **Searches** | **Results** |
| --- | --- | --- |
| 1 | (voluntary or charity or charities or charitable or "third sector" or TSO or "civic sector" or "social sector" or "social enterprise" or Non Government* or NGO or "community based" or "community sector" or "community organisation" or "profit" or "nonprofit" or "not for profit" or "big society" or "civil society" or foundation*).ti,ab. | 13726 |
| 2 | charities/ | 513 |
| 3 | voluntary groups/ or community groups/ or neighbourhood care/ or third sector/ or voluntary organisations/ | 1301 |
| 4 | 1 or 2 or 3 | 14348 |
| 5 | ((us* adj2 evidence) or (role adj2 evidence) or (role adj2 research) or (us* adj2 research)).ti,ab. | 2265 |
| 6 | ((knowledge or research or evidence) adj1 (transfer* or exchange or broker* or practice or mobilisation or translation or translating or generation or utilization or utilisation or implementation or dissemination)).ti,ab. | 858 |
| 7 | ((uptake or "take up" or adoption or adopting or adopted or intergrat*) adj3 (research or evidence or knowledge or science)).ti,ab. | 253 |
| 8 | evidence based practice/ or evidence based policy/ | 2487 |
| 9 | Research implementation/ | 222 |
| 10 | decision making/ and (knowledge or evidence or research).ti,ab. | 1723 |
| 11 | "what works".ti,ab. | 243 |
| 12 | (diffus* adj2 (innovation or research or evidence or knowledge or information)).ti,ab. | 64 |
| 13 | 5 or 6 or 7 or 8 or 9 or 10 or 11 or 12 | 6999 |
| 14 | 4 and 13 | 284 |

Database: Social Policy and Practice (SPP)

Host: OVID

Data Parameters: 201401

Date Searched: 10/03/2014

| **#** | **Searches** | **Results** |
| --- | --- | --- |
| 1 | (voluntary or charity or charities or charitable or "third sector" or TSO or "civic sector" or "social sector" or "social enterprise" or Non Government* or NGO or "community based" or "community sector" or "community organisation" or "profit" or "nonprofit" or "not for profit" or "big society" or "civil society" or foundation*).ti,ab. | 24211 |
| 2 | ((us* adj2 evidence) or (role adj2 evidence) or (role adj2 research) or (us* adj2 research)).ti,ab. | 2659 |
| 3 | ((knowledge or research or evidence) adj1 (transfer* or exchange or broker* or practice or mobilisation or translation or translating or generation or utilization or utilisation or implementation or dissemination)).ti,ab. | 941 |
| 4 | ((uptake or "take up" or adoption or adopting or adopted or intergrat*) adj3 (research or evidence or knowledge or science)).ti,ab. | 264 |
| 5 | "what works".ti,ab. | 1274 |
| 6 | (diffus* adj2 (innovation or research or evidence or knowledge or information)).ti,ab. | 33 |
| 7 | 2 or 3 or 4 or 5 or 6 | 4967 |
| 8 | 1 and 7 | 395 |

Database: CommunityWise

Host: Oxmill

Data Parameters: unkown

Date Searched: 05/03/2014

| # | Searches | Results |
| --- | --- | --- |
| 1 | Using evidence | 93 |
| 2 | Use of evidence | 34 |
| 3 | Evidence use | 3 |
| 4 | What works and (charity or voluntary or third sector or profit) | 20 |
| 5 | Role of research | 19 |

Database: ASSIA

Host: ProQuest

Data Parameters: (1987 - current)

Date Searched: 10/03/2014

Set#: S1

Searched for: (voluntary or charity or charities or charitable or "third sector" or TSO or "civic sector" or "social sector" or "social enterprise" or Non Government* or NGO or "community based" or "community sector" or "community organisation" or "profit" or "nonprofit" or "not for profit" or "big society" or "civil society" or foundation*)

Databases: Applied Social Sciences Index and Abstracts (ASSIA)

Results: 26316*

Set#: S2

Searched for: ((us* N/2 evidence) or (role N/2 evidence) or (role N/2 research) or (us* N/2 research))

Databases: Applied Social Sciences Index and Abstracts (ASSIA)

Results: 11194*

Set#: S3

Searched for: ((knowledge or research or evidence) N1 (transfer* or exchange or broker* or practice or mobilisation or translation or translating or generation or utilization or utilisation or implementation or dissemination))

Databases: Applied Social Sciences Index and Abstracts (ASSIA)

Results: 7°

Set#: S4

Searched for: ((uptake or "take up" or adoption or adopting or adopted or intergrat*) N3 (research or evidence or knowledge or science))

Databases: Applied Social Sciences Index and Abstracts (ASSIA)

Results: 56°

Set#: S5

Searched for: "what works"

Databases: Applied Social Sciences Index and Abstracts (ASSIA)

Results: 359°

Set#: S6

Searched for: (diffus* N2 (innovation or research or evidence or knowledge or information))

Databases: Applied Social Sciences Index and Abstracts (ASSIA)

Results: 0°

Set#: S7

Searched for: s2 or s3 or s4 or s5 or s6

Databases: Applied Social Sciences Index and Abstracts (ASSIA)

Results: 11604*

Set#: S8

Searched for: s1 and s7

Databases: Applied Social Sciences Index and Abstracts (ASSIA)

Results: 661°

* Duplicates are removed from your search, but included in your result count.

° Duplicates are removed from your search and from your result count.

Database: British Library Social Welfare Profile

Host: <http://socialwelfare.bl.uk/>

Date Searched: 10/03/2014

| # | Search Terms | Search located/included |
| --- | --- | --- |
| 1. | "evidence use" | 5/1 |
| 2. | "using evidence" | 119/6 |
| 3. | "role of evidence" | 20/0 |
| 4. | "research use" | 23/0 |
| 5. | "use of research" | 66/0 |
| 6. | “using research” | 67/1 |
| 7. | "knowledge mobilisation" | 5/3 |
| 8. | "knowledge transfer" | 107/7 |
| 9. | "what works" AND charit* | 9/1 |
| 10. | "what works" AND "third sector" | 0/0 |
| 11. | "what works" AND "not for profit" | 2/0 |
